# Heterozygous variant in *FGFR3* underlying severe phenotypes in the second trimester: a case report

**DOI:** 10.1186/s12920-023-01517-8

**Published:** 2023-04-19

**Authors:** Shujun Chen, Hongmei Dong, Yong Luo, Yingpin Zhang, Pan Li

**Affiliations:** 1 Department of Ultrasound, The First People’s Hospital of Chongqing Liang Jiang New Area, Chongqing, 400010 China; 2 Department of Ultrasound, The Second Affiliated Hospital of Chongqing Medical University, Chongqing, 400010 China; 3 Institute of Ultrasound Imaging of Chongqing Medical University, Chongqing, 400010 China; 4 Department of Ultrasound, Chongqing Health Center for Women and Children, Women and Children’s Hospital of Chongqing Medical University, Chongqing, 400010 China

**Keywords:** Achondroplasia, *FGFR3* gene, Heterozygous, Rare variant, Severe phenotype

## Abstract

**Background:**

Achondroplasia is a congenital skeletal system malformation caused by missense variant of *FGFR3* gene with an incidence of 1 per 20,000–30,000 newborns, which is an autosomal dominant inheritance disease. Despite similar imaging features, the homozygous achondroplasia is absolutely lethal due to thoracic stenosis, whereas heterozygous achondroplasia does not lead to fetal death.

**Case presentation:**

A fetus with progressive rhizomelic short limbs and overt narrow chest was detected by prenatal ultrasound in the second trimester. Gene sequencing results of amniotic fluid sample indicated a rare missense variant NM_000142.4: c.1123G > T(p.Gly375Cys), leading to a glycine to cysteine substitution. Re-sequencing confirmed that it was a heterozygous variant, and thoracic stenosis was then confirmed in the corpse by radiological examination.

**Conclusions:**

We identified a heterozygous variant of the *FGFR3* gene as the rare pathogenic variant of severe achondroplasia in a fetus. Heterozygous variants of p.Gly375Cys may have a severe phenotype similar to homozygote. It’s crucial to combine prenatal ultrasound with genetic examination to differentiate heterozygous from homozygous achondroplasia. The p.Gly375Cys variant of *FGFR3* gene may serve as a vital target for the diagnosis of severe achondroplasia.

## Background

Achondroplasia is a congenital skeletal system malformation caused by missense variant of *FGFR3* gene with an incidence of 1 per 20,000–30,000 newborns, which is an autosomal dominant inheritance disease [1–3]. Variants in the *FGFR3* gene lead to hyperactivation of tyrosine kinase, promoting multiple mitosis, such as carcinogenesis and overgrowth of skin, but inhibiting the proliferation and terminal differentiation of chondrocytes. This paradoxical phenomenon may be due to the activation of defense mechanisms that protect mammals from cancer. Because chondrocytes are the most intense target of the *FGFR3* gene, the activation of defense mechanisms is particularly severe during chondrocyte activity (this has been thoroughly elaborated in references 1, 2, and 5). The study conducted by Di Rocco F in 2014 demonstrated that *FGFR3* greatly affects both endochondral and intramembranous ossification, resulting in impaired development of craniofacial bones and stunted growth as the main clinical manifestations. Therefore, it affects both endochondral and intramembranous ossification [4, 5].

The phenotypic features of affected individuals include disproportionate short stature, rhizomelic shortening of the arms, a prominent forehead, midface hypoplasia, large skull roof, small skull base and spinal cord compression. Additionally, homozygous achondroplasia is absolutely lethal due to thoracic stenosis. Whereas heterozygous achondroplasia does not lead to fetal death. Radiologic images of the skull, spine, chest, and extremities reveal these characteristic features [1, 3, 6]. In this report, we describe a case of heterozygous achondroplasia with thoracic stenosis, which was diagnosed in the second trimester based on ultrasound features and genetic testing.

## Case presentation

The case is of term female baby delivered by a gravida 2, parity 0(G2P0) at week 25, who was 29 years old and had an early miscarriage with unknown aetiology. The parents were healthy without family history of genetic diseases or history of infection and medication during the pregnancy. Prenatal ultrasound was firstly performed at 12 + 3w gestational age (GA), the thickness of nuchal translucensy was 0.13 cm and the crown-rump length was in accorded with the clinical gestational week. Non invasice prenatal genetic testing showed a low-risk gestation. At 19w GA, short fetal limbs were found by routine ultrasonography with the femur below -3SD. The biparental and fetal chromosome examination and whole-exon sequencing were then recommended. Ultrasonographic features at 22w GA indicated obviously short limbs, rhizomelic shortening of the hummers, the femur/abdominal circumference and femur/plantar length, which suggested pathogenic skeletal dysplasia. At 24 + 5w GA, the long bones of fetal limbs were obviously short and the condition was progressively aggravated (Tables 1 and 2). Narrow chest was found with a ratio of chest/abdominal circumference less than 0.89. The fetus was finally diagnosed with suspected achondroplasia by ultrasonography.

**Table 1:** 19w, 22w, 24 + 5w ultrasound essential biological data and standard deviation (SD)

GA	BPD	HC	AC	FL	HUM
19w	49 (+ 0.93SD)	179 (+ 0.24SD)	155 (+ 0.50SD)	22.0 (− 3.55SD)	22.1 (− 1.81SD)
22w	57 (+ 1.41SD)	196 (− 0.47SD)	160 (− 0.91SD)	27.1 (− 3.77SD)	27.3 (− 1.79SD)
24 + 5w	65 (+ 1.35SD)	231 (− 0.10SD)	205 (+ 0.13SD)	28.5 (− 5.70SD)	27.8 (− 2.82SD)

**Table 2:** 22w, 24 + 5w US extensional biological data

GA	Plantar length	Chest circumference	Femur/abdominal circumference	Femur/plantar length	Chest/abdominal circumference
22w	37.2	145	0.169	0.73	0.91
24 + 5w	45.5	147	0.139	0.63	0.68

**Fig. 1:**
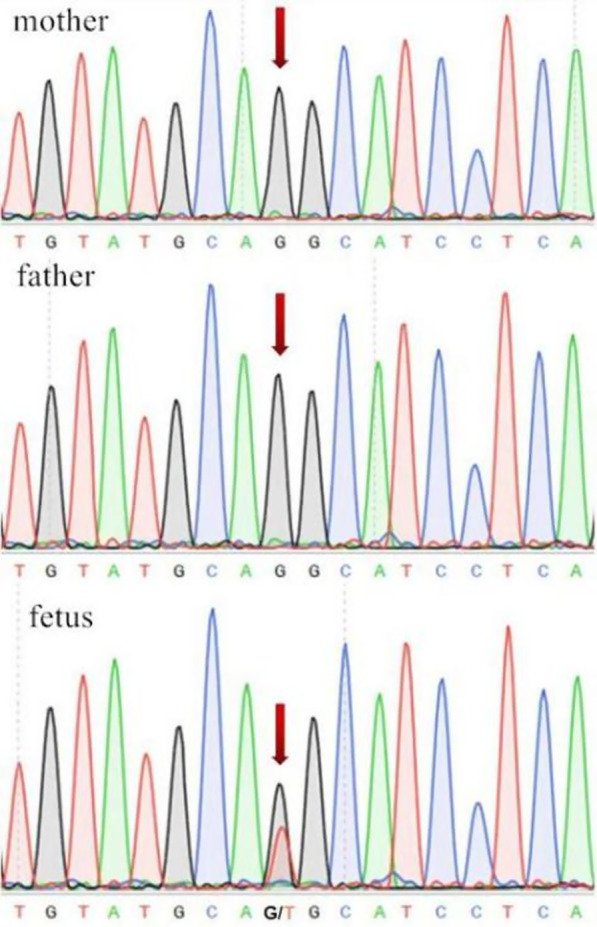
Sanger sequencing chromatograms showing a missense variant c.1123G > T in the affected fetus in comparison to her unaffected parents

No significant abnormalities were found in biparental and fetal chromosomes. Since heterozygous achondroplasia with a similarly severe phenotype has never been reported previously, we then collected parental blood and fetal amniotic fluid exfoliated cells to perform whole exome sequencing of *FGFR3* gene. As shown in Fig. 1, the sequencing results indicated that a single-base changed from G-to-T at codon 375, which caused a glycine to be replaced by a cysteine.

**Fig. 2:**
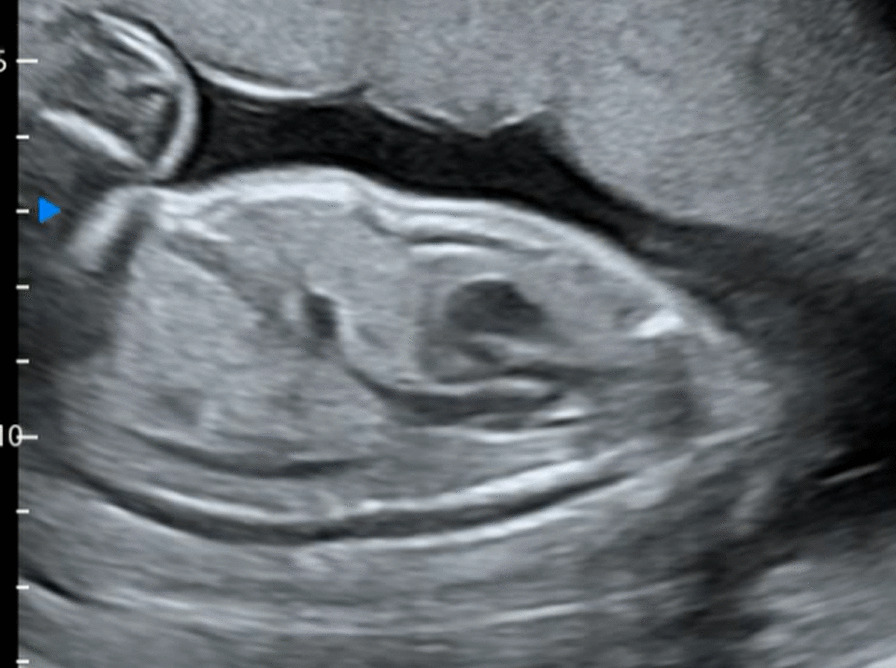
The sagittal image of narrow chest at 24 + 5w GA

**Fig. 3:**
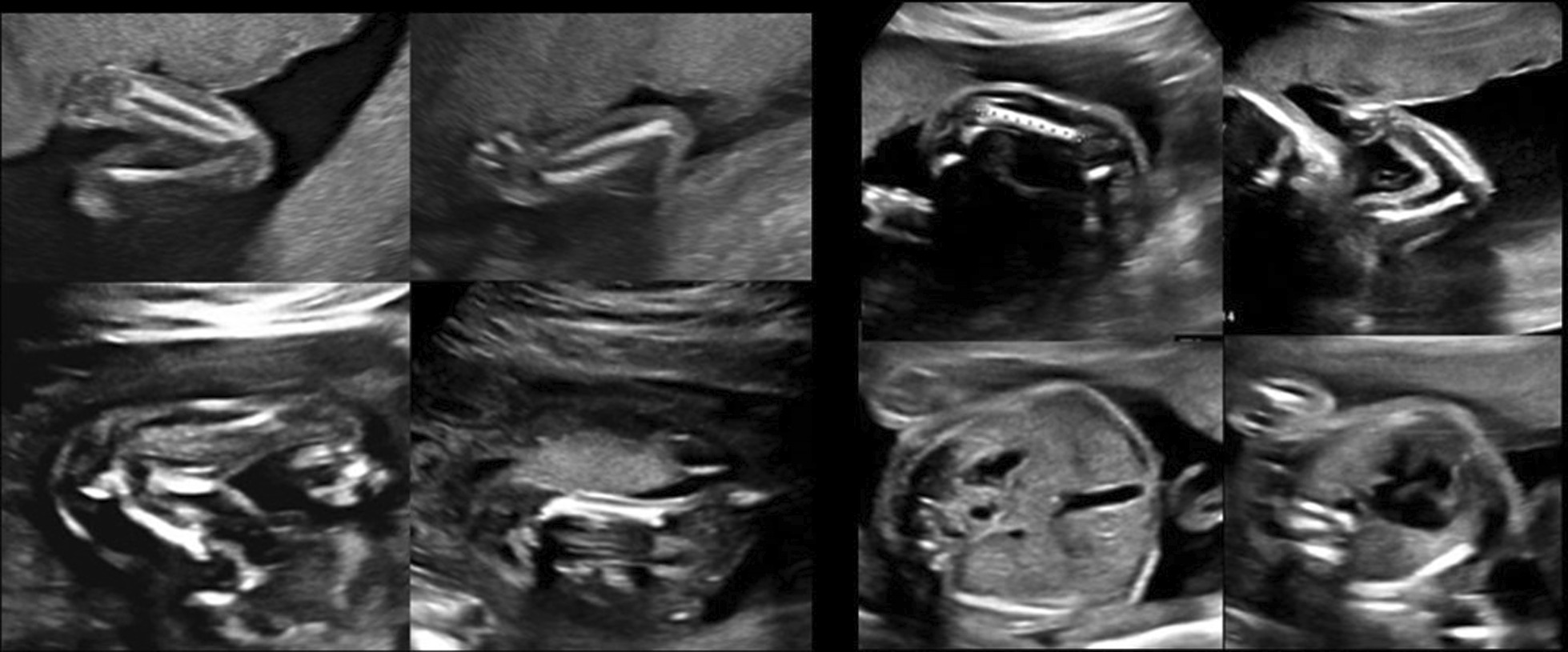
Transverse section of narrow chest and short limbs at 22w GA and 24 + 5w GA

**Fig. 4:**
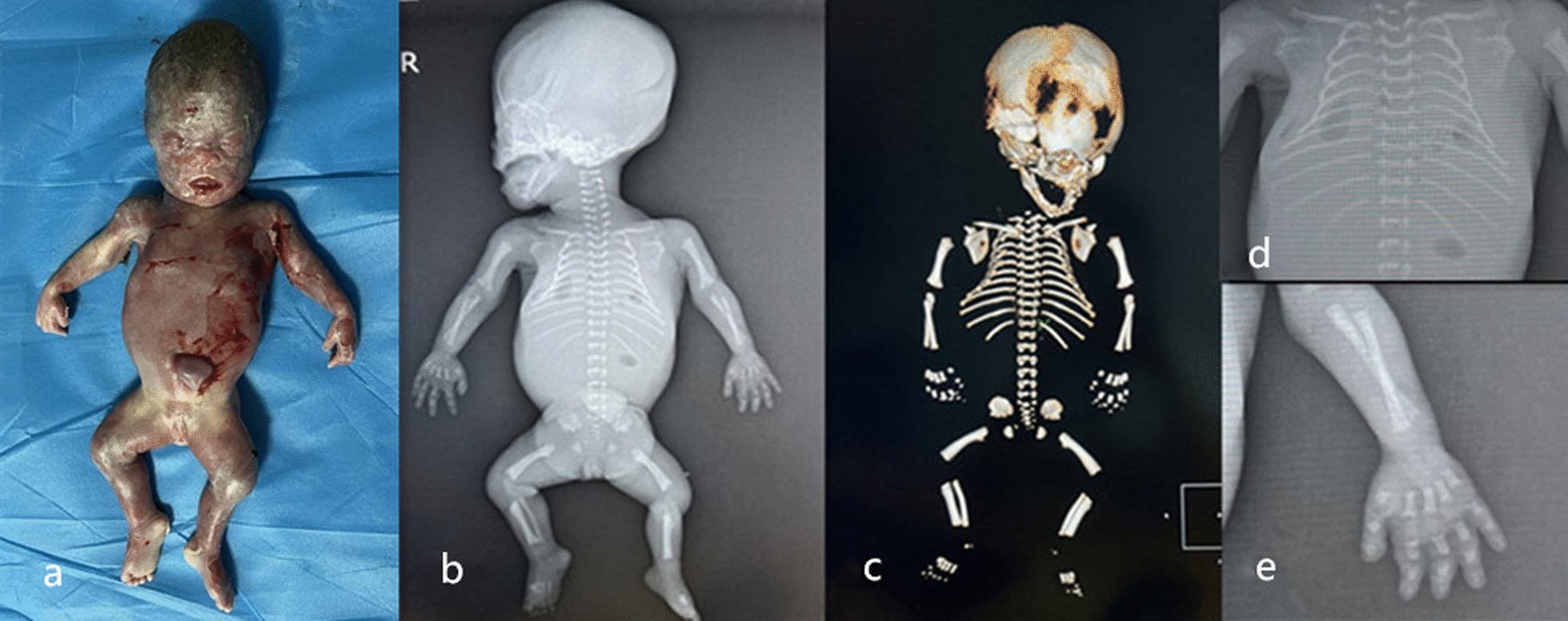
Physiological characteristics and radiographic evidence of the corpse. **a** Physiological characteristics of the corpse: disproportionate shortening of long bones, frontal bossing, midface hypoplasia, and protuberant abdomen, talipes equinovarus in the right side. **b** X-ray image showing disproportionate shortening of long bones, large skull roof and small skull base. **c** Computerized tomography 3D bone reconstruction showing narrow thoracic shape as a bell, flat midface and spine. **d** X-ray image showing narrow chest and flat vertebrae. **e** the trident hand

Given the genetic test reports and ultrasound features (Figs. 2 and 3), this case was finally diagnosed with severe achondroplasia. After prenatal consultation, the couple requested to terminate the pregnancy. The physiological characteristics and radiographic evidence of the corpse (Fig. 4) confirmed the final diagnosis.

## Discussion

**Fig. 5:**
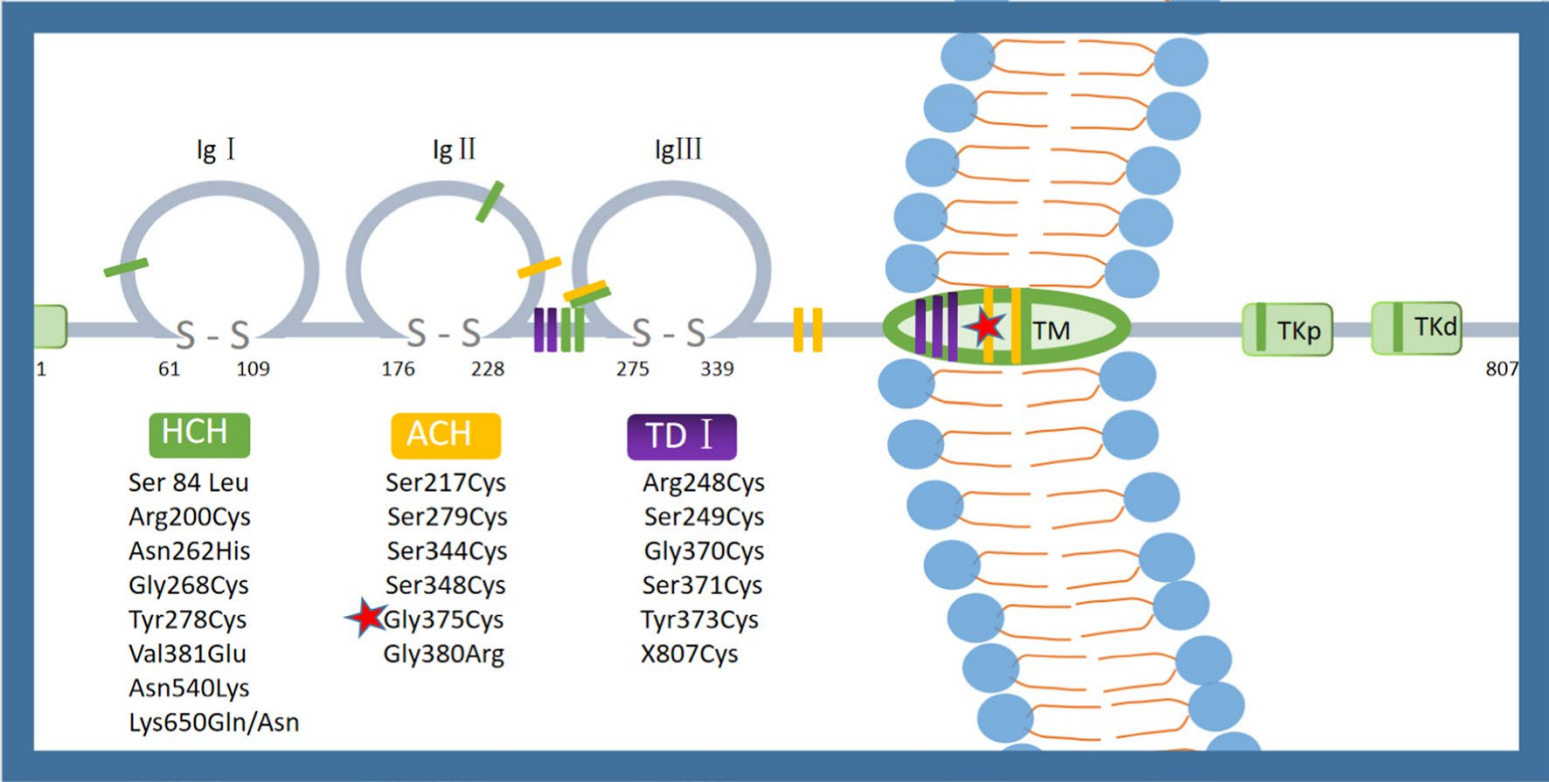
Topology map of *FGFR3* with major sites of variant. *ACH* achondroplasia; *TDI* thanatophoric dysplasia type I.; *HYP* hypochondroplasia; *TKp*/*d* proximal and distal tyrosine kinase domains; *TM* transmembrane

The *FGFR3* (fibroblast growth factor receptor 3) have an extracellular ligand-binding domain, a transmembrane domain and an intracellular domain that contains a split tyrosine kinase subdomain [1, 2]. Variants in the *FGFR3* gene at different locations result in varying degrees of skeletal deformities, including thanatophoric dysplasia, achondroplasia, and hypochondroplasia (ordered by severity). Thanatophoric dysplasia is typically characterized by a cloverleaf skull, an extremely narrow thorax, or long bones that are extremely short and curved. The phenotypic features of affected achondroplasia individuals include disproportionate short stature, rhizomelic shortening of the arms, a prominent forehead, midface hypoplasia, large skull roof, small skull base and spinal cord compression. In contrast, the symptoms of hypochondroplasia are usually milder, and shortened femur is only occasionally detected prenatally. We have summarized the variant sites that have been reported to cause thanatophoric dysplasia, achondroplasia, or hypochondroplasia (Fig. 5). 98% of achondroplasia patients are caused by the variants of p.Gly380Arg in *FGFR3*, while the remaining 1% is attributed to other variants. Based on the current reports of achondroplasia, we found none of the heterozygotes showed thoracic stenosis according to the phenotypic analysis. Three cases were reported to be caused by the variant of p.Gly375Cys, but the phenotype was only described in two of them [7–9]. These two patients were diagnosed at two years old and four days after birth respectively. They shared typical imaging features and vertebral flattening, but did not have apparent narrow chests [8, 9].

Heuertz S. suggested that the cysteine residues in the extracellular domain can cause excess disulfide bond formation, leading to a tertiary structure change of *FGFR3*, further activating tyrosine kinase, resulting in more severe phenotypes [10]. This mechanism may be one of the possible causes of the severe phenotype in this case, as the variant of p.Gly375Cys (indicated by the red star in Fig. 5) also creates additional cysteine residues.

Traditionally, the diagnosis of achondroplasia is based on genetic examination and radiological features [2]. Prenatal ultrasound serve as a routine repeatable imaging method providing additionally valuable information. Although the surviving achondroplasia fetuses have a low life satisfaction due to abnormal appearance and progressive spinal pain [11, 12], some families are still willing to accept such children with mild symptoms who are expected to have a nearly normal lifespan with short femurs in the third trimester. Therefore, accurate prenatal diagnosis and risk assessment of achondroplasia are important.

In conclusion, it is crucial to combine prenatal ultrasound with genetic examination to fully evaluate the severe phenotype of heterozygous achondroplasia, and the variant of p.Gly375Cys may serve as a vital target for the diagnosis.

## Data Availability

All data generated or analysed during this study are included in this published article.

## Abbreviations

*FGFR3*: Fibroblast growth factor receptor 3
GA: Gestational age
TDI: Thanatophoric dysplasia type I
